# Tools for Identifying Potentially Inappropriate Prescriptions for Children and Their Applicability in Clinical Practices: A Systematic Review

**DOI:** 10.3389/fphar.2022.787113

**Published:** 2022-05-18

**Authors:** Siyu Li, Liang Huang, Zhe Chen, Linan Zeng, Hailong Li, Sha Diao, Zhi-Jun Jia, Guo Cheng, Qin Yu, Lingli Zhang

**Affiliations:** ^1^ Department of Pharmacy, West China Second University Hospital, Sichuan University, Chengdu, China; ^2^ Evidence-based Pharmacy Center, West China Second University Hospital, Sichuan University, Chengdu, China; ^3^ Key Laboratory of Birth Defects and Related Diseases of Women and Children, Ministry of Education, Chengdu, China; ^4^ West China School of Medicine, Sichuan University, Chengdu, China; ^5^ West China School of Pharmacy, Sichuan University, Chengdu, China; ^6^ Laboratory of Molecular Translational Medicine, Center for Translational Medicine, Sichuan University, Chengdu, China; ^7^ Department of Pediatrics, West China Second University Hospital, Sichuan University, Chengdu, China; ^8^ National Drug Clinical Trial Institute, West China Second University Hospital, Sichuan University, Chengdu, China

**Keywords:** children, adolescents, inappropriate prescriptions, potentially inappropriate medication, potential prescribing omission, rational drug use, adverse drug reaction

## Abstract

**Background:** Drug use safety in children is a global public health problem. The potentially inappropriate prescription screening tools are expected to reduce adverse drug reactions and promote rational drug use.

**Objectives:** To systematically evaluate children’s potentially inappropriate prescription screening tools and validation studies on these tools.

**Methods:** We systematically searched six databases PubMed, Embase, Cochrane Library, CNKI, VIP and Wanfang Data. Two reviewers independently selected articles by the eligible criteria and extracted data. Then we evaluated the coverage of diseases or drugs in these tools and the consistency of items between tools.

**Results:** Five children’s potentially inappropriate prescription screening tools were identified, most tools were formed by Delphi expert consensus and focused on respiratory system drugs, anti-infective drugs, and gastrointestinal drugs. The coincidence rates of items between the POPI and the POPI Int, the POPI and the POPI United Kingdom, the POPI United Kingdom and the POPI int, and the POPI United Kingdom and the PIPc were 82.0, 55.1, 51.0 and 2.2% respectively, and the KIDs List did not overlap other four tools. Only the POPI tool developed by French experts was comprehensively validated by studies and most tools have not been validated.

**Conclusion:** The development of screening tools for potentially inappropriate prescribing in children is a neglected field and most tools lack studies to validate clinical applicability. More researchers need to form their national potentially inappropriate prescription screening tools for children based on the best available clinical evidence and the actual clinical situation in their countries.

## Introduction

The safety of drug use in children is a global public health problem. About 125 children worldwide die every day due to inappropriate drug use (Ye, 2019). However, the study results have shown that adverse drug reactions (ADRs) in about 15.2% pediatric patients could be entirely avoided, and 9.1% could be partly avoided. Not taking into account a history of allergy or altered renal function and not respecting the recommended dose were the most frequent causes of entirely avoidable ADRs (Jonville-Béra et al., 2009).

In 2011, PubMed introduced the term “Inappropriate Prescribing” in its Medical Subject Headings (MeSH) list (NCBI, 2011). Inappropriate prescribing is a prescribing behavior with risks greater than benefits for patients, which can lead to higher mortality, more hospitalizations and ADRs. Potentially inappropriate prescription (PIP) is the prescription whose potential risk is higher than general prescriptions and more likely to be judged as inappropriate after adequate assessment by clinical pharmacists or clinicians, and usually the drugs in PIPs can be replaced by other safer and more effective drugs(O'Connor et al., 2012; Fu et al., 2018). PIP includes two parts: potentially inappropriate medication (PIM), patients have medication indications but the risk of adverse events after drug use may be greater than the benefits (e.g., when tetracycline is used in children under 8 years, its bone and tooth toxicity may outweigh its benefit); potential prescribing omission (PPO), in the absence of contraindications, omission to prescribe drugs that are significantly beneficial to the patient’s life expectancy or quality (e.g., oral rehydration solution is not given to children with diarrhea who do not receive intravenous fluids). (spinewine et al., 2007; Beers, 1991).

Currently, identifying PIPs in adults (especially in the elderly) by a series of tools, is relatively common. In 2014, Kaufmann conducted a systematic review of inappropriate prescribing identifying tools for adults and collected 46 published tools (Kaufmann et al., 2014). Among these tools, the Beers criteria (Beers, 1991) proposed by an American geriatrics expert Beers in 1991 and the STOPP/START criteria (Gallagher et al., 2008) developed by experts from the Cork University Hospital in Ireland in 2008 are the most widely used (Gillespie et al., 2013; Hill-Taylor et al., 2016; Huang et al., 2020; Lopez-Rodriguez et al., 2020). The randomized controlled trial results (Gillespie et al., 2013; Hill-Taylor et al., 2016; Gallagher et al., 2011; Dalleur et al., 2014; Frankenthal et al., 2014; O'Connor et al., 2016) have shown that using PIP screening tools as an intervention to identify PIPs in the elderly could effectively improve the rationality of drug treatment and significantly reduce PIMs, ADR, falls, hospital length-of-stay, re-admission, and medication costs.

The current methods to analyze potentially inappropriate prescriptions in patients can be classified into two categories, explicit and implicit methods (Spinewine et al., 2007). Explicit methods are focused on measuring how prescriptions fit a set of predefined criteria on drugs (e.g., Beers and STOPP/START criteria for elderly patients) and will be updated according to the available evidence. The implicit criteria are based on the subjective comprehensive assessment of a health professional, which takes into account the overall situation of the patient and whether the prescription corresponds to an indication or need (Lopez-Rodriguez et al., 2020). The medication appropriateness index (MAI) (Hanlon et al., 1992) is the most accepted implicit method internationally. The biggest difference between explicit and implicit tools is that explicit tools require relatively less clinical experience and knowledge of users and the assessment results are more objective (Curtin et al., 2019).

Compared with adults, the development and clinical use of children’s PIP screening tools have not been extensively studied. Our study aimed to systematically evaluate children’s PIP screening tools and validation studies on these tools.

## Methods

### Search Strategy

We searched six databases Pubmed, Embase, Cochrane Library, CNKI, Wanfang Data, VIP. Then we read the references of included articles and relevant reviews as a supplementary search. The search strategy was adjusted specifically for each database and included a combination of medical subject headings and free text terms for (“child” or “pediatrics” or “infant” or “adolescent”) and (“high risk medications” or “high risk prescriptions” or “potentially inappropriate drugs” or “potentially inappropriate medications” or “potentially inappropriate prescriptions” or “omission prescriptions” or “POPI” or “PIPc”). The deadline for all retrieval was May 2021.

### Eligible Criteria

The following studies were included: 1) PIP screening tools, described as a tool used to identify potentially dangerous or known ineffective prescription patterns, described as a tool used to identify prescription patterns that do not conform to best clinical practices or current guidelines or described as a tool for identifying prescription patterns that are easily ignored or omitted; 2) validation studies on PIP screening tools, described as a clinical study which aims to assess the feasibility or reliability of PIP screening tools; 3) Target population: children (0–18 years).

The following studies were excluded: 1) repeated publication, 2) review, 3) unobtainable full-texts, 4) not the latest version, 5) non-Chinese and non-English.

### Literature Selection and Data Extraction

Two reviewers (SL and ZC) independently screened all titles and abstracts to determine potential eligible literature. Then they applied the eligibility criteria to perform the final selection by reading the full text. When different opinions occurred between reviewers, they would discuss and make the final decision. If they could not reach an agreement, the final decision would be made by a third reviewer (LH).

Two reviewers (SL and LH) independently extracted data from included studies and cross-checked them. The extracted data included: 1) the basic information of articles (the first author, published year, title, published country, etc.); 2) the basic characteristics of PIP screening tools (name, development method, healthcare setting, target population, etc.); 3) all items of each PIP screening tool; 4) the basic information of validation studies (year, country, healthcare setting, PIP screening tool name, prevalence of PIM and PPO in children, etc.).

### Data Analysis

We used descriptive statistical analysis methods to evaluate the children’s PIP screening tools, including counting the coverage of diseases or drugs and the number of items in these tools, and calculating the coincidence rate of items between tools $\left(Coincidence\;rate=\frac{Number\;of\;same\;items\times 2}{Number\;of\;all\;items\;in\;compared\;tools}\right)$ . These results were presented in tables.

## Results

### Literature Search and Selection

**FIGURE 1:**
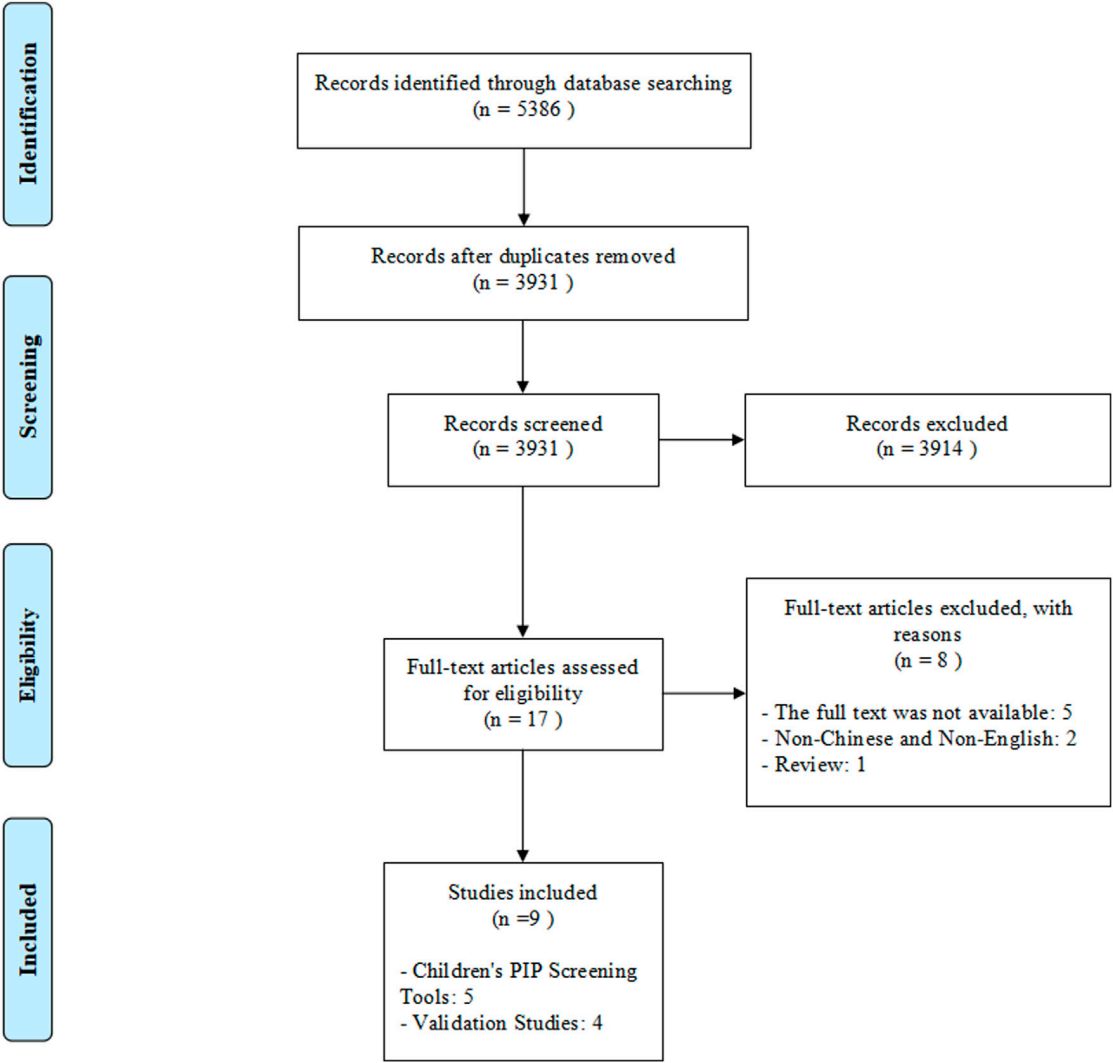
Flow diagram of the study selection process.

A total of 5,386 records were retrieved through database searching and no additional records were identified through reading the references of included articles and relevant reviews. After removing duplicates, 3,931 articles were initially selected by screening titles and abstracts. Then removing 3,914 irrelevant articles, 17 articles were assessed for eligibility at full-text reading. Finally, five children’s PIP screening tools and four validation studies were included in this systematic review. (Figure 1).

### Characteristics of the Children’s PIP Screening Tools

A total of five children’s PIP screening tools were identified, the POPI tool (Prot-Labarthe et al., 2014) developed by French experts, the PIPc tool (Barry et al., 2016) developed by British and Irish experts, the POPI United Kingdom tool (Corrick et al., 2019) established by British experts through modifying French POPI tool, the POPI Int tool (Sadozai et al., 2020) formed by the international expert group consensus based on French POPI tool and the KIDs List (Meyers et al., 2020) published by American experts. All tools were explicit tools. Except the PIPc was mainly used in primary health care to detect PIPs in children ≤16 years old, other tools unlimited the healthcare setting where they were used and were suitable for children ≤18 years old. Different from the other three tools, using the PIPc and the KIDs List hardly needed clinical information such as children’s disease conditions. (Table 1).

**TABLE 1:** Basic characteristics of children’s PIP screening tools.

Author	Year	Country	Name	Development method	Healthcare setting	Need patient clinical information (such as disease status, etc.)	Target population
Sonia Prot-Labarthe et al	2014	France	POPI	Delphi consensus	Unlimited	Necessary	18 years or younger
Emma Barry et al	2016	United Kingdom and Ireland	PIPc	Delphi consensus	Primary health care	Unnecessary	16 years or younger
Fenella Corrick et al	2017	United Kingdom	POPI United Kingdom	Modifying the POPI tool	Unlimited	Necessary	18 years or younger
Laily Sadozai et al.	2020	Multinational consensus	POPI Int	Modifying the POPI tool + Delphi consensus	Unlimited	Necessary	18 years or younger
Rachel S Meyers et al	2020	America	KIDs List	Establishing an expert group	Unlimited	Unnecessary	18 years or younger

Except the POPI United Kingdom tool which was directly formed by modifying the POPI tool based on the British formulary and clinical guidelines, an expert group was involved in the development of tools and the Delphi consensus technique was mostly used. The classification of items in the POPI tool and its development process were similar to the STOPP/START tool for the elderly (Gallagher et al., 2008). In these two tools, researchers constructed their criteria based on physiological systems (classification of PIP according to the physiological system of the disease covered by the prescription), and both included two modules, PIM and PPO. And both further screened and revised the preliminary criteria through Delphi consensus before forming the final criteria. Moreover, the development process of the KIDs List was more similar to general evidence-based clinical guidelines. First, an expert group was established, then relevant clinical evidence was retrieved on the topic and expert opinions were integrated. Finally, the GRADE method was used to grade the quality of evidence and form a recommendation strength for each item.

### Comparison of Children’s PIP Screening Tools

The POPI tool (Prot-Labarthe et al., 2014) had 105 items, which was the tool with the largest number of items and covered the most extensive physiological systems and health problems, including the digestive system, respiratory system, skin problems, nervous system, urinary system, and other problems (pain and fever, mosquito repellent, vitamin supplement and antibiotic prophylaxis). Except the KIDs List (Meyers et al., 2020) which only involved the PIM part, other tools all contained two parts of PIM and PPO. (Table 2).

**TABLE 2:** Comparison of children’s PIP screening tools.

Name	Number of items	Covered health problems	Types of PIPs								
			PIMs								PPOs
			Drug choice	Dosage	Duration	Route of administration	Drug-disease interaction	Drug-drug interaction	Drug-food interaction	Over-prescribing	Under-prescribing
POPI	105 (PIM 80, PPO 25)	Digestive system, respiratory system, skin problems, nervous system, urinary system, and other problems (pain and fever, mosquito repellent, vitamin supplement, and antibiotic prophylaxis)	**√**	**√**	**√**	**√**	**√**	**√**	**√**	**√**	**√**
PIPc	12 (PIM 7, PPO 5)	Digestive system, respiratory system, skin problems, nervous system	**√**					**√**			**√**
POPI United Kingdom	80 (PIM 60, PPO 20)	Digestive system, respiratory system, skin problems, nervous system, urinary system, and other problems (pain and fever, vitamin supplement, and antibiotic prophylaxis)	**√**	**√**	**√**	**√**	**√**		**√**	**√**	
POPI Int	73 (PIM 58, PPO 15)	Digestive system, respiratory system, skin problems, nervous system, urinary system, and other problems (pain and fever, mosquito repellent, vitamin supplement, and antibiotic prophylaxis)	**√**	**√**	**√**	**√**	**√**			**√**	**√**
KIDs List	77 (PIM 77)^a^	Digestive system, respiratory system, skin problems, nervous system, cardiovascular system, and other problems (anti-microbial infection)	**√**	**√**	**√**						

a The KIDs List includes 67 drugs and 10 pharmaceutical excipients.

PIPs, potentially inappropriate prescriptions; PPOs, potential prescribing omissions; PIMs, potentially inappropriate medications.

Both the POPI Int tool and the POPI United Kingdom tool were formed based on the POPI tool. The POPI Int tool only deleted items that experts believed were not generic across countries from the POPI (e.g., “Oral solutions of ibuprofen administered in more than three doses per day using a graduated pipette of 10 mg/kg” and “Nitrofurantoin used as a prophylactic in children with urinary infections” were deleted), and no other changes had been made. Therefore, all items in the POPI Int were completely derived from the POPI, and the coincidence rate of the two tools was 82.0%. Except to delete items in the POPI that were not supported by the British formulary and clinical guidelines, the POPI United Kingdom also modified the content of some items to make them more suitable for British children. In the end, the POPI United Kingdom tool retained 80 items in the POPI, of which 51 items were unchanged and 29 items were modified. The coincidence rate of the POPI United Kingdom and the POPI was 55.1% and the coincidence rate of the POPI United Kingdom and the POPI Int was 51.0%. The POPI, POPI United Kingdom, and POPI Int had the highest degree of overlap in children’s PIP screening items on the digestive system conditions (vomiting, gastroesophageal reflux) and respiratory system conditions (asthma, bronchiolitis, bronchial infection). For example, they all believed that “Prescribing metoclopramide, domperidone, H_2_-receptor antagonist (long-term use) to children with nausea, vomiting or gastroesophageal reflux”, “Loperamide for the treatment of invasive diarrhea” and “Loperamide for children under 3 years old” were potentially inappropriate prescriptions.

Comparing the PIPc tool with other tools, we found that it overlapped POPI UK’s with only one item (“Loperamide for children under four” was a potentially inappropriate prescription for children), and the coincidence rate was 2.2%. This tool mainly focused on identifying PIPs in children with respiratory diseases (especially asthma), and six of 12 items were on this topic.

The KIDs List did not overlap the other four tools. In general, the coincidence rates of children’s PIP screening tools were not very high.

### Validation Studies on Children’s PIP Screening Tools

At present, the clinical empirical studies of the POPI tool in France have evaluated its clinical applicability (whether can sensitively detect PIPs in pediatric populations) (Berthe-Aucejo et al., 2019a) and the reproducibility of results among users (consistency of assessment results for the same prescription among different users) (Berthe-Aucejo et al., 2019b), and these results were published in 2019. These study results showed that the POPI tool had good clinical applicability and good reproducibility of results among users, and it could detect children’s potentially inappropriate prescriptions in French clinical practice. However, the number of studies was small, with only one each, and these studies were conducted only in France. So it was not clear whether it could be well used to detect children’s PIPs in other countries. (Table 3).

**TABLE 3:** Validation studies on children’s PIP screening tools.

Name	Healthcare setting	Number of children	Number of prescriptions	Prevalence of PIMs	Prevalence of PPOs
POPI	Emergency department	15,973	18,562	2.9%	2.3%
	Community pharmacy	2,225	4,780	12.3%	6.1%
PIPc	Primary health care	414,856	414,856	3.5%	2.5%
POPI United Kingdom	Emergency department and inpatient setting	400	—	—	—

PIPs, potentially inappropriate prescriptions; PPOs, potential prescribing omissions; PIMs, potentially inappropriate medications.


$$Prevalence\;of\;PIMs=\frac{Number\;of\;prescriptions\;with\;PIM\geq 1}{Number\;of\;prescriptions}$$

$$Prevalence\;of\;PPOs=\frac{Number\;of\;prescriptions\;with\;PPO\geq 1}{Number\;of\;prescriptions}$$

The clinical applicability and the reproducibility of results among users of the PIPc tool needed to be further evaluated. Currently, only one published study evaluated the PIPc tool used to identify children’s PIPs in primary health care (with limited clinical information for children). The study results (Barry et al., 2018) showed that a single item had a great influence on the screening results of PIMs and PPOs. When the item “should be prescribed spacers for children with asthma (PPO)” was included, the PPO prevalence rose from 2.5 to 11.5%. And when the item “Carboxysteine should not be prescribed to children (PIM)" was deleted, the PIM prevalence dropped from 3.5 to 0.29%. Moreover, the study results also showed that the tool might not be as well applicable to primary health care where the clinical information was inadequate as the researchers envisioned. (Table 3).

The POPI United Kingdom, POPI Int and KIDs List currently did not have the published study results on their clinical applicability and reproducibility of results among users (The POPI United Kingdom had a clinical applicability study that was only published as an abstract (Corrick et al., 2017) and the study results were not fully presented). The applicability and reliability of screening tools have not been validated by studies, which may be an important reason that hinders their clinical application.

## Discussion

### Comparison With PIPs Screening Tools for Adults

This systematic review identified five potentially inappropriate prescription screening tools for children and the number was far less than adults (Kaufmann et al., 2014). Most PIP screening tools were developed by the Delphi expert consensus and were used to promote clinical rational drug use and reduce medication risk. Unlike the elderly PIP screening tools (Beers criteria and STOPP/START criteria, etc.) that focus on cardiovascular and nervous system drugs, children’s PIP screening tools pay more attention to respiratory drugs, anti-infective drugs and gastrointestinal drugs. The clinical applicability and the reliability of PIP screening tools for the elderly, such as the STOPP/START criteria, have been confirmed in many countries (Gallagher et al., 2009), while most children’s PIP screening tools lack studies to validate their clinical applicability and reproducibility of results among users. This may be the reason that hinders the further clinical use of these tools. To be consistent with the current best clinical practice and evidence, the Beers criteria is updated every 3 years. However, there is no regular update mechanism for children’s PIP screening tools.

### International Transferability of Children’s PIP Screening Tools

Corrick F et al.(Corrick et al., 2019) pointed out that the POPI tool might not be directly applicable to other countries including the United Kingdom, when they evaluated and modified the POPI tool to form the POPI United Kingdom that is more suitable for British practice. Because some drugs mentioned in the POPI tool did not be marketed in other countries and some items might be contradictory to the guidelines and formulary of other countries. In their article, Corrick F et al.(Corrick et al., 2019) indicated that since there were currently no endemic insect-borne diseases in the United Kingdom, they directly deleted all items on the topic of “mosquitoes” in POPI, which were considered not applicable to the practice in the United Kingdom. Besides, some items in POPI were removed, which were inconsistent with the British guidelines and formulary [e.g., the POPI regarded “nitrofurantoin treatment for children younger than six″ as a children’s PIP, which was not in line with NICE guideline CG54 (urinary tract infection: nitrofurantoin is recommended to use nitrofurantoin for children 3 months and older)].

When reviewing and evaluating these children’s PIP screening tools, we found that they could not be directly used for Chinese children. The same reasons were that some items in these tools contradict the current clinical practice in China or some medicines have not yet been marketed in China, such as “pimozide” and “prochlorperazine” in the KIDs List and “palivizumab” in the POPI have not been marketed in China. Moreover, the POPI tool classified “use ibuprofen oral solution 10 mg/kg for children with fever or pain, more than three times a day (the maximum daily dose over 30 mg/kg/d)” as a potentially inappropriate prescription for children. However, the “WHO model formulary for children in 2010” (WHO, 2010) and the “China National Formulary for Children in 2013” (China National Formulary Editorial Committee, 2013) believed that “ibuprofen oral solution does not exceed 4 times per day, the maximum daily dose of 40 mg/kg/d" was reasonable. We suggest that the existing children’s PIP screening tools should be modified based on the actual conditions in each country, which makes tools more suitable for clinical practice in different countries, instead of directly using the existing tools. This may be a more appropriate choice.

### Urgency of Forming Suitable Children’s PIP Screening Tools

The limited clinical studies in children (Smyth, 2001; Smyth, 2016) lead to the lack of information on children’s medication (Angoulvant et al., 2010), which further induces off-label drug use in children (Zhang et al., 2013; Gore et al., 2017; Nir-Neuman et al., 2018). Moreover, children may receive drug prescriptions from prescribers with varying degrees of pediatric experience and knowledge in different healthcare settings (including private clinics, emergency departments, pediatric wards of general hospitals and children’s specialist hospitals). So, rational drug use in children is facing enormous challenges. The use of PIP screening tools is expected to effectively reduce PIM and ADR and promote the clinical rational use of drugs, which has been confirmed in the elderly (Hill-Taylor et al., 2016). In addition, children’s PIP screening tools can also quantify and compare the incidence of children’s PIPs among different prescribers, medical institutions and regions, which supports government departments to take relevant and effective measures to improve the reasonable rate of prescriptions.

### Limitations

It is worth mentioning that this study also has some limitations: first, the study results might have language bias. Our study only included PIP screening tools published in Chinese or English and ignored some tools published in other languages. Second, the latest version of the French POPI tool was the 2016 version (Prot-Labarthe et al., 2016), but this version was only published in French. So we had to include the 2014 English version of POPI. It should be noted that some items in the 2016 version might differ from the 2014 version.

Corrick F et al. also conducted a systematic review on children’s PIP screening tools in 2019 (Corrick et al., 2020) and obtained three tools, POPI, PIPc and POPI United Kingdom. Their systematic review mainly elaborated on the development methods of each tool rather than comparing the differences of items in these tools like our study. Our study can be considered as a supplement and an update to the study conducted by Corrick F.

## Conclusion

The development of screening tools for identifying potentially inappropriate prescribing in children is a neglected field and most tools lack studies to validate clinical applicability. More researchers need to form their national potentially inappropriate prescription screening tools for children based on the best available clinical evidence and the actual clinical situation in their countries. Moreover, screening tools need to be validated in time to promote clinical use, thereby improving rational drug use and reducing medication risk in children.

## Data Availability

The original contributions presented in the study are included in the article/Supplementary Material, further inquiries can be directed to the corresponding authors.

## References

[B1] Angoulvant F. Kaguelidou F. Dauger S. Alberti C. (2010). Fewer Infants Than Older Patients in Paediatric Randomised Controlled Trials. Eur. J. Epidemiol. 25 (8), 593–601. 10.1007/s10654-010-9480-2 20563834

[B2] Barry E. Moriarty F. Boland F. Bennett K. Smith S. M. (2018). The PIPc Study-Application of Indicators of Potentially Inappropriate Prescribing in Children (PIPc) to a National Prescribing Database in Ireland: a Cross-Sectional Prevalence Study. BMJ OPEN 8 (10), e022876. 10.1136/bmjopen-2018-022876 PMC6196813 30344174

[B3] Barry E. O'Brien K. Moriarty F. Cooper J. Redmond P. Hughes C. M. (2016). PIPc Study: Development of Indicators of Potentially Inappropriate Prescribing in Children (PIPc) in Primary Care Using a Modified Delphi Technique. BMJ OPEN 6 (9), e012079. 10.1136/bmjopen-2016-012079 PMC5020844 27601499

[B4] Beers M. H. (1991). Explicit Criteria for Determining Inappropriate Medication Use in Nursing Home Residents. UCLA Division of Geriatric Medicine. Arch. Intern Med. 151 (9), 1825–1832. 10.1001/archinte.151.9.1825 1888249

[B5] Berthe-Aucejo A. Nguyen N. P. K. K. Angoulvant F. Boulkedid R. Bellettre X. Weil T. (2019b). Interrater Reliability of a Tool to Assess Omission of Prescription and Inappropriate Prescriptions in Paediatrics. Int. J. Clin. Pharm. 41 (3), 734–740. 10.1007/s11096-019-00819-1 30972535

[B6] Berthe-Aucejo A. Nguyen P. K. H. Angoulvant F. Bellettre X. Albaret P. Weil T. (2019a). Retrospective Study of Irrational Prescribing in French Paediatric Hospital: Prevalence of Inappropriate Prescription Detected by Pediatrics: Omission of Prescription and Inappropriate Prescription (POPI) in the Emergency Unit and in the Ambulatory Setting. BMJ OPEN 9 (3), e19186. 10.1136/bmjopen-2017-019186 PMC6475152 30898791

[B7] China National Formulary Editorial Committee (2013). China National Formulary (Children's Edition) Chemical and Biological Products Volume. China: People's Military Medical Publishing House.

[B8] Corrick F. Choonara I. Conroy S. Sammons H. (2019). Modifying a Paediatric Rational Prescribing Tool (POPI) for Use in the UK. Healthc. (Basel) 7 (1), 33. 10.3390/healthcare7010033 PMC6473318 30791668

[B9] Corrick F. Conroy S. Sammons H. Choonara I. (2020). Paediatric Rational Prescribing: A Systematic Review of Assessment Tools. Int. J. Environ. Res. Public Health 17 (5). 10.3390/ijerph17051473 PMC7084281 32106497

[B10] Corrick F. J. Conroy S. Choonara I. Sammons H. (2017). Developing Paediatric Rational Prescribing Criteria. Arch. Dis. Child. 102 (Suppl. 1), A84. 10.1136/archdischild-2017-313087.209

[B11] Curtin D. Gallagher P. F. O'Mahony D. (2019). Explicit Criteria as Clinical Tools to Minimize Inappropriate Medication Use and its Consequences. Ther. Adv. Drug Saf. 10, 584371383. 10.1177/2042098619829431 PMC6378636 30800270

[B12] Dalleur O. Boland B. Losseau C. Henrard S. Wouters D. Speybroeck N. (2014). Reduction of Potentially Inappropriate Medications Using the STOPP Criteria in Frail Older Inpatients: a Randomised Controlled Study. Drugs Aging 31 (4), 291–298. 10.1007/s40266-014-0157-5 24566877

[B13] Frankenthal D. Lerman Y. Kalendaryev E. Lerman Y. (2014). Intervention with the Screening Tool of Older Persons Potentially Inappropriate Prescriptions/screening Tool to Alert Doctors to Right Treatment Criteria in Elderly Residents of a Chronic Geriatric Facility: a Randomized Clinical Trial. J. Am. Geriatr. Soc. 62 (9), 1658–1665. 10.1111/jgs.12993 25243680

[B14] Fu M. Y. Wang Zh. F. Ma Y. Y. (2018). Review of International Assessment Indicators of Rational Drug Use. Chin. Pharm. Aff. 32 (04), 538–545. 10.16153/j.1002-7777.2018.04.019

[B15] Gallagher P. Baeyens J. P. Topinkova E. Madlova P. Cherubini A. Gasperini B. (2009). Inter-rater Reliability of STOPP (Screening Tool of Older Persons' Prescriptions) and START (Screening Tool to Alert Doctors to Right Treatment) Criteria Amongst Physicians in Six European Countries. AGE AGEING 38 (5), 603–606. 10.1093/ageing/afp058 19435757

[B16] Gallagher P. F. O'Connor M. N. O'Mahony D. (2011). Prevention of Potentially Inappropriate Prescribing for Elderly Patients: a Randomized Controlled Trial Using STOPP/START Criteria. Clin. Pharmacol. Ther. 89 (6), 845–854. 10.1038/clpt.2011.44 21508941

[B17] Gallagher P. Ryan C. Byrne S. Kennedy J. O'Mahony D. (2008). STOPP (Screening Tool of Older Person's Prescriptions) and START (Screening Tool to Alert Doctors to Right Treatment). Consensus Validation. Int. J. Clin. Pharmacol. Ther. 46 (2), 72–83. 10.5414/cpp46072 18218287

[B18] Gillespie U. Alassaad A. Hammarlund-Udenaes M. Mörlin C. Henrohn D. Bertilsson M. (2013). Effects of Pharmacists' Interventions on Appropriateness of Prescribing and Evaluation of the Instruments' (MAI, STOPP and STARTs') Ability to Predict Hospitalization-Aanalyses from a Randomized Controlled Trial. PLOS ONE 8 (5), e62401. 10.1371/journal.pone.0062401 23690938 PMC3656885

[B19] Gore R. Chugh P. K. Tripathi C. D. Lhamo Y. Gautam S. (2017). Pediatric Off-Label and Unlicensed Drug Use and its Implications. Curr. Clin. Pharmacol. 12 (1), 18–25. 10.2174/1574884712666170317161935 28322168

[B20] Hanlon J. T. Schmader K. E. Samsa G. P. Weinberger M. Uttech K. M. Lewis I. K. (1992). A Method for Assessing Drug Therapy Appropriateness. J. Clin. Epidemiol. 45 (10), 1045–1051. 10.1016/0895-4356(92)90144-c 1474400

[B21] Hill-Taylor B. Walsh K. A. Stewart S. Hayden J. Byrne S. Sketris I. S. (2016). Effectiveness of the STOPP/START (Screening Tool of Older Persons' Potentially Inappropriate Prescriptions/Screening Tool to Alert Doctors to the Right Treatment) Criteria: Systematic Review and Meta-Analysis of Randomized Controlled Studies. J. Clin. Pharm. Ther. 41 (2), 158–169. 10.1111/jcpt.12372 26990017

[B22] Huang Y. Zhang L. Huang X. Liu K. Yu Y. Xiao J. (2020). Potentially Inappropriate Medications in Chinese Community-Dwelling Older Adults. Int. J. Clin. Pharm. 42 (2), 598–603. 10.1007/s11096-020-00980-y 32026350

[B23] Jonville-Béra A. P. Saissi H. Bensouda-Grimaldi L. Beau-Salinas F. Cissoko H. Giraudeau B. (2009). Avoidability of Adverse Drug Reactions Spontaneously Reported to a French Regional Drug Monitoring Centre. Drug Saf. 32 (5), 429–440. 10.2165/00002018-200932050-00006 19419237

[B24] Kaufmann C. P. Tremp R. Hersberger K. E. Lampert M. L. (2014). Inappropriate Prescribing: a Systematic Overview of Published Assessment Tools. Eur. J. Clin. Pharmacol. 70 (1), 1–11. 10.1007/s00228-013-1575-8 24019054

[B25] Lopez-Rodriguez J. A. Rogero-Blanco E. Aza-Pascual-Salcedo M. Lopez-Verde F. Pico-Soler V. Leiva-Fernandez F. (2020). Potentially Inappropriate Prescriptions According to Explicit and Implicit Criteria in Patients with Multimorbidity and Polypharmacy. MULTIPAP: A Cross-Sectional Study. PLOS ONE 15 (8), e237186. 10.1371/journal.pone.0237186 PMC7423095 32785232

[B26] Meyers R. S. Thackray J. Matson K. L. McPherson C. Lubsch L. Hellinga R. C. (2020). Key Potentially Inappropriate Drugs in Pediatrics: The KIDs List. J. Pediatr. Pharmacol. Ther. 25 (3), 175–191. 10.5863/1551-6776-25.3.175 32265601 PMC7134587

[B27] NCBI (2011). Inappropriate Prescribing-MeSH-NCBI. Available: https://www.ncbi.nlm.nih.gov/mesh/?term=inappropriate+prescription (Accessed July 10, 2021).

[B28] Nir-Neuman H. Abu-Kishk I. Toledano M. Heyman E. Ziv-Baran T. Berkovitch M. (2018). Unlicensed and Off-Label Medication Use in Pediatric and Neonatal Intensive Care Units: No Change over a Decade. Adv. Ther. 35 (7), 1122–1132. 10.1007/s12325-018-0732-y 29949042

[B29] O'Connor M. N. Gallagher P. O'Mahony D. (2012). Inappropriate Prescribing: Criteria, Detection and Prevention. Drugs Aging 29 (6), 437–452. 10.2165/11632610-000000000-00000 22642779

[B30] O'Connor M. N. O'Sullivan D. Gallagher P. F. Eustace J. Byrne S. O'Mahony D. (2016). Prevention of Hospital-Acquired Adverse Drug Reactions in Older People Using Screening Tool of Older Persons' Prescriptions and Screening Tool to Alert to Right Treatment Criteria: A Cluster Randomized Controlled Trial. J. Am. Geriatr. Soc. 64 (8), 1558–1566. 10.1111/jgs.14312 27365262

[B32] Prot-Labarthe S. Weil T. Angoulvant F. Boulkedid R. Alberti C. Bourdon O. (2014). POPI (Pediatrics: Omission of Prescriptions and Inappropriate Prescriptions): Development of a Tool to Identify Inappropriate Prescribing. PLOS ONE 9 (6), e101171. 10.1371/journal.pone.0101171 24978045 PMC4076280

[B33] Prot-Labarthe S. Weil T. Nguyen N. P. Berthe-Aucejo A. Angoulvant F. Boulkedid R. (2016). Consensus Validation of a Tool to Identify Inappropriate Prescribing in Pediatrics (POPI). Arch. Pediatr. 23 (5), 481–490. 10.1016/j.arcped.2016.02.010 27067037

[B34] Sadozai L. Sable S. Le Roux E. Coste P. Guillot C. Boizeau P. (2020). International Consensus Validation of the POPI Tool (Pediatrics: Omission of Prescriptions and Inappropriate Prescriptions) to Identify Inappropriate Prescribing in Pediatrics. PLOS ONE 15 (10), e240105. 10.1371/journal.pone.0240105 PMC7535059 33017423

[B35] Smyth R. L. (2016). Getting Paediatric Clinical Trials Published. LANCET 388 (10058), 2333–2334. 10.1016/S0140-6736(16)32125-0 27845080

[B36] Smyth R. L. (2001). Research with Children. Paediatric Practice Needs Better Evidence-Ggained in Collaboration with Parents and Children. BMJ 322 (7299), 1377–1378. 10.1136/bmj.322.7299.1377 11397728 PMC1120459

[B37] Spinewine A. Schmader K. E. Barber N. Hughes C. Lapane K. L. Swine C. (2007). Appropriate Prescribing in Elderly People: How Well Can it Be Measured and Optimised? LANCET 370 (9582), 173–184. 10.1016/S0140-6736(07)61091-5 17630041

[B38] WHO (2010). WHO Model Formulary for Children 2010. World Health Organization. Available at: https://apps.who.int/iris/handle/10665/44309 (Accessed July 21, 2021)

[B39] Ye Q. Y. (2019). Discussion and Countermeasures of Children's Drug Safety. China's health Ind. 16 (02), 152–153. 10.16659/j.cnki.1672-5654.2019.02.152

[B40] Zhang L. Li Y. Liu Y. Zeng L. Hu D. Huang L. (2013). Pediatric Off-Label Drug Use in China: Risk Factors and Management Strategies. J. Evid. Based Med. 6 (1), 4–18. 10.1111/jebm.12017 23557523

